# Hepato-protective effects of *Glossogyne tenuifolia* in Streptozotocin-nicotinamide-induced diabetic rats on high fat diet

**DOI:** 10.1186/s12906-019-2529-1

**Published:** 2019-06-06

**Authors:** Shibu Marthandam Asokan, Ruoh-Yuh Wang, Tsu-Han Hung, Wan-Teng Lin

**Affiliations:** 1 Department for Management of Science and Technology Development, Ton Duc Thang University, Ho Chi Minh City, Vietnam; 2 Faculty of Applied Sciences, Ton Duc Thang University, Ho Chi Minh City, Vietnam; 3 Department of Nutritional Science, Fu-Jen Catholic University, New Taipei City, Taiwan; 4 Nutrition Department, Far Eastern Memorial Hospital, New Taipei City, Taiwan; 5 Department of Hospitality Management, College of Agriculture, Tunghai University, No.1727, Sec4, Taiwan Boulevard, Xitun, District, Taichung, 40704 Taiwan, Republic of China

**Keywords:** *Glossogyne tenuifolia* (GT), Hepatoprotective, Diabetes mellitus (DM), High-fat diet (HFD)

## Abstract

**Background:**

*Glossogyne tenuifolia* (GT) is a traditional herbal tea in Penghu Island, Taiwan. Its extract is traditionally been used as an antipyretic, hepatoprotective and anti-inflammatory remedy in folk medicine among local residents. The present study investigated whether GT could improve streptozotocin-induced acute liver injury of type 2 diabetes mellitus.

**Methods:**

Male Wistar rats aged eight weeks were induced to be hyperglycemic by the subcutaneous injection of streptozotocin-nicotinamide (STZ-NA) and a combination of a high-fat diet (HFD) (N group). The animals were given GT extracts at a low dose (50 mg/kg) (L group) or a high dose (150 mg/kg) (H group) or an anti-diabetic drug (acarbose) (P group) in drinking water for 4 weeks.

**Results:**

The results revealed that STZ-NA increased hepatomegaly, hepatocyte cross-sectional area, hypertrophy-related pathways (IL6/STAT3-MEK5-ERK5, NFATc3, p38 and JNK MAPK), proapoptotic molecules (cytochrome C, cleaved caspase-3), and fibrosis-related pathways (FGF-2, pERK1/2). These pathway components were then expressed at lower levels in the L and H group when compared with the N group. The liver-protective effect of GT in STZ-NA-induced diabetic rats with hyperlipidemia was through an enhancement in the activation of the compensatory PI3K-Akt and Bcl2 survival-related pathway.

**Conclusion:**

The results demonstrate that the hot water extracts of GT efficiently ameliorates the STZ-NA-induced diabetes associated liver damage in rat models.

## Background

Diabetes mellitus (DM) is a chronic metabolic disease with high prevalence rate that has raised global public health concern. Type 2 DM (T2D) in particular, is characterized by a progressive reduction in insulin sensitivity correlated with the dysfunction of pancreatic β-cells [1, 2]. The resulting hyperglycemia, a major feature of DM, causes oxidative stress and various organ damages leading to disorders such as diabetic retinopathy, diabetic nephropathy, cardiovascular diseases and other endocrine disorders [2–4]. Further, the role of oxidative stress is a crucial contributing factors for complications associated with diabetes [5].

Most among the numerous risk factors of non-alcoholic fatty liver disease (NAFLD) causes metabolic imbalances or insulin resistance to trigger the development of diabetes [6–9]. Furthermore, NAFLD is also known to develop into chronic liver disease in obese individuals [10]. Hepatic-apoptosis is of the common event responsible for NAFLD-induced liver injury [11]. Unregulated incidence of apoptosis is also followed by hepatic-fibrosis [11, 12]. As there is no effective therapeutic drug in treating NAFLD, diet control and diet associated approach are the viable options to prevent NAFLD at present. Therefore, inhibition of hepatic-apoptosis is a prospective strategy in the treatment for NAFLD.

The common drugs currently used for DM treatment cause heavy economic burden and serious concerns on their side effects [13]. Therefore, efficient but cost-effective herbal remedies that do not show any long-term side effects are highly desired. Therapeutic potentials of several herbal teas or Chinese herbal medicines known to be abundant sources of antioxidants, particularly phenolics and antioxidant vitamins, potentially display hypoglycemic effects [14, 15]. In addition, extracts from a variety of such herbs or plants can also potentially avert drug-induced acute liver failure due to their antioxidant potential. *Glossogyne tenuifolia* (GT) Cassini (Hsiang Ju grass) a common perennial herb distributed around the accessible coastal areas of Penghu Island, Taiwan is known for its liver protective effects [16–18].

Extracts of GT are traditionally been used to prepare herbal drink that provide protection against sunstroke but there is a long history of it being used as a remedy in the local Folk medicine for their antipyretic, hepatoprotective and anti-inflammatory effects [15, 16, 19–23]. Administration of GT extract in Streptozotocin (STZ)–nicotinamide (NA)-induced diabetic rats have shown to inhibit α-glucosidase in vitro and enhance anti-oxidative capacity and hypoglycemic effect [15, 24]. Several bioactive principles in GT extracts have been identified and shown to provide both anti-inflammatory and anti-viral effects with respect to production of signaling mediators, like TNF-alpha, IL-6 and IFN-gamma, in human blood [25, 26]. Luteolin and luteolin-7-glucoside in GT is known to enhance free radical scavenging capacity to act against atherosclerosis and inhibits HCC liver cancer cell lines [26]. However, there is still no adequate scientific evidence on the biological functions of GT as a nutraceutical health drink.

In a quest to find effective and novel drugs to prevent and treat DM, various experimental animal models of diabetes are used. STZ-NA-induced is one of the suitable model for drug screening against diabetes [27]. STZ is administered to exert cytotoxic effects on pancreatic β-cells and NA is administered to provide a certain degree of protection against excessive deterioration of pancreatic function. The model resemble closer to effects associated with T2DM in terms of responsiveness of insulin to glucose levels.

Therefore, the present study evaluated the hepatoprotective effect of GT extracts against STZ-NA and HFD-induced hepatotoxicity in mice.

## Methods

### Preparation of GT extract

The GT herbal material used in the experiment was received from the Kaohsiung District Agricultural Improvement Station in Penghu, Taiwan, ROC; the specimens were confirmed by Dr. Tamilselvi Shanmugam, China medical University hospital, Taiwan; and the specimens (Voucher Number 106030802WTL) were deposited in the herbarium of Thung Hai University. GT was extracted and characterized as previously described [15, 16]. Briefly, 50 g of dried GT was homogenized and boiled in 500 mL of deionized water for 1 h. The contents were centrifuged and the supernatant was filtered, and the residues were collected and mixed with 500 mL water and the extraction was repeated again. The extracts were pooled and concentrated to dry powder using vacuum freeze-dryer. The yield rates of the extracts by hot water were similar to those previously reported.

### HPLC conditions for analyzing GT herbal extract

The extracts were characterized by reverse phase chromatography analysis in a Inertsil ODS-3 column fitted with a precolumn (20 X 3.9 mm) using High performance liquid chromatography with an ultraviolet–visible detector (Shimadzu, Kyoto, Japan) equipped with Shimadzu LC-20AT reciprocating pumps. Linear gradient elution was carried with 100% methanol as a mobile phase and 1% acetic acid (in water) with a flow rate of 1 mL/min. The elution was detected by monitoring the eluent at 350 nm.

### Animal experiments

Eight weeks old male Wistar rats were procured from BioLASCO Taiwan Co., Ltd. for the study. The animals were maintained in proper cages at 22 °C ± 1 °C, 60% ± 10% RH, with a 12 h/12 h day/night alternation and fed ad libitum with a laboratory chow (#5001, PMI Feeds, Inc., Brentwood, MO, USA) for a week before the experiments begun. T1DM was induced by subcutaneous injection of 65 mg/kg (in 10 mM ice-cold citrate buffer, pH 4.5) streptozotocin (STZ, Sigma, St. Louis, MO) after subcutaneous injection of nicotinamide (NA, 230 mg/kg) (pH 4.5). Following STZ injection the animals were changed to a high fat-containing diet (40% energy from fat) and subsequently another dose of NA and STZ was injected after 24 h. Meanwhile the control group rats received subcutaneous injections of citrate buffer alone (control group). After a week, the fasting blood glucose levels in blood samples collected from tail vein were determined and the rats that showed above 220 mg/dL of blood glucose were considered as DM. The rats were randomly segregated in different groups (*n* = 9) and named as control group, Non-treated DM group, low-dose (50 mg/kg) of GT extract treated DML group, high-dose (150 mg/kg) of GT extract treated HML group and DM with acarbose (known drug for controlling blood sugar level) treated DMA group. The GT powder and 20 mg/kg of acarbose were diluted in deionized drinking water for 4 weeks. The drinking water was prepared freshly by considering the volume consumed on the previous day. The body weight changes were noted every week and the amount of food and water intake was noted every day. Any changes in the animal behavior and activity were recorded. The animals were euthanized by terminal anesthesia with isoflurane followed by decapitation. All animal experimental protocols were reviewed and approved by the Animal Experimental Committee of Fu Jen University (IACUC No. A10056), and the study was conducted in accordance with the principles of laboratory animal care [28].

### Plasma biochemical analysis

Blood samples were collected in centrifuge tubes containing 1000 IU mL^− 1^ heparin and the plasma glucose, insulin, (triglycerides) TG and High-density lipoprotein (HDL) cholesterol levels using commercially available assay kits.

### Histological evaluation of liver sections

Liver tissues were fixed in 10% formalin were then embedded in paraffin and cut into 4 μm thick tissue slices. Slides were hydrated by immersing in series of graded alcohols (100, 95, and 75%), for 10–15 min each. The liver sections were then stained using hematoxylin and eosin (H&E) and with Masson’s trichrome. The histological changes were observed using light microscope equipped attached with a CCD camera (BX-51, Olympus, Tokyo, Japan).

### Tissue protein extraction and western blotting

Liver protein extracts were obtained by homogenizing the liver tissue in lysis buffer (0.05 M Tris-HCl, pH 7.4, 0.15 M NaCl, 0.25% deoxycholic acid, 1% NP-40, 1 mM EDTA). The homogeneates were centrifuged at 13,000 rpm for 30 min and the supernatants were collected and stored for further use. Western blot analysis was performed following methods reported previously [29]. The protein concentrations were determined by the Bradford protein assay method. Proteins were resolved using 8–12% SDS-PAGE and were then immunoblotted using an enhanced chemiluminescence assay (ECL; Perkin Elmer Life Science, Inc., USA), and images were recorder in a Fuji LAS-4000.

### Statistical analysis

All data are represented as mean ± SEM. To evaluate the statistical differences were analyzed using a one-way ANOVA with the Statistical Analysis System (SAS Institute, Cary, NC, USA). *P* < 0.05 was considered statistically significant.

## Results

### Characterization of GT extracts and effect of GT extracts on diabetic rats

**Fig. 1:**
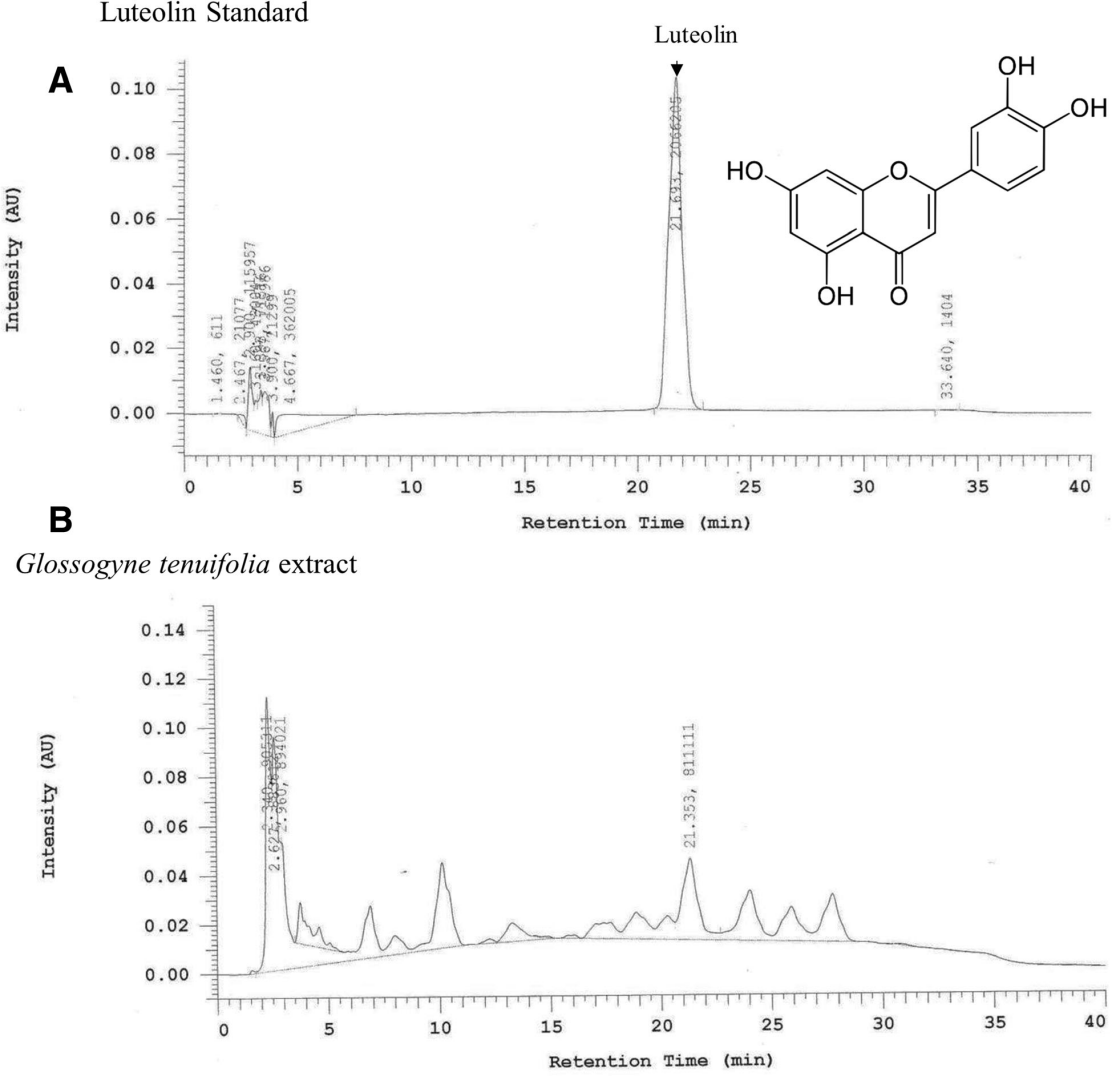
Characterization of *Glossogyne tenuifolia* extract: HPLC analysis of *G*.*tenuifolia* show the presence of Luteolin. **a**. HPLC Spectrum of standard luteolin and structure of luteolin and the structure of luteolin. **b**. HPLC analysis of *G*.*tenuifolia* showing presence of luteolin spectrum

**Fig. 2:**
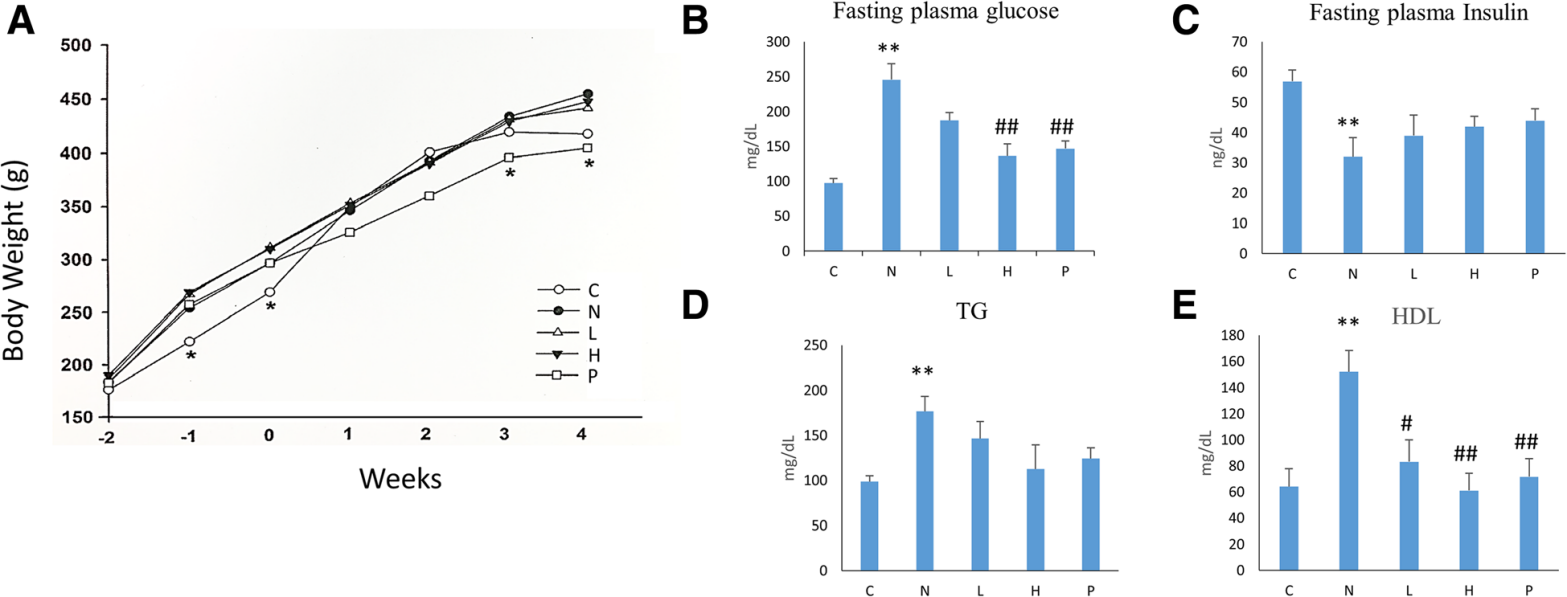
Effects of different doses of *G*.*tenuifolia* extracts on diabetic rats. Changes in (**a**) body weight, (**b**) fasting plasma, (**c**) fasting insulin, (**d**) triglyceride (TG) (**e**) High-density lipoprotein (HDL) cholesterol. Data represent mean ± SEM. C: control; N: STZ-NA + HFD; L: *Glossogyne tenuifolia* low dose (50 mg/kg); H: *Glossogyne tenuifolia* high dose (150 mg/kg); P: acarbose (positive control). **p < 0.01 compared with the C group; #*P* < 0.05 and ##*P* < 0.01 when compared with the N group

The GT extracts were characterized based on comparative retention time observed in HPLC analysis. Retention time of luteolin was observed as 21.6 min (Fig. 1a) and HPLC analysis of GT extracts showed same retention time (Fig. 1b). Further, administration of GT extracts in high fat diet fed diabetic rats did not show any apparent secondary effects nor showed any significant difference in the body weight when compared to control rats (Fig. 2a). However the fasting plasma glucose levels were significantly high in the STZ-NA administered rats which was regulated to that of the normal levels in the treatment groups (Fig. 2b). Meanwhile the fasting plasma insulin levels were significantly reduced in the STZ-NA administered rats whereas treatment rats showed an increasing trend in the plasma insulin levels (Fig. 2c). However high fat diet fed diabetic rats showed other symptoms of NAFLD as seen from the elevated plasma TG and HDL levels (Fig. 2d, e) but the levels in the rats treated with high and low doses of GT and in those treated with acarbose remained similar to that of the normal controls.

### GT extract administration alleviated STZ-NA-induced effects

**Fig. 3:**
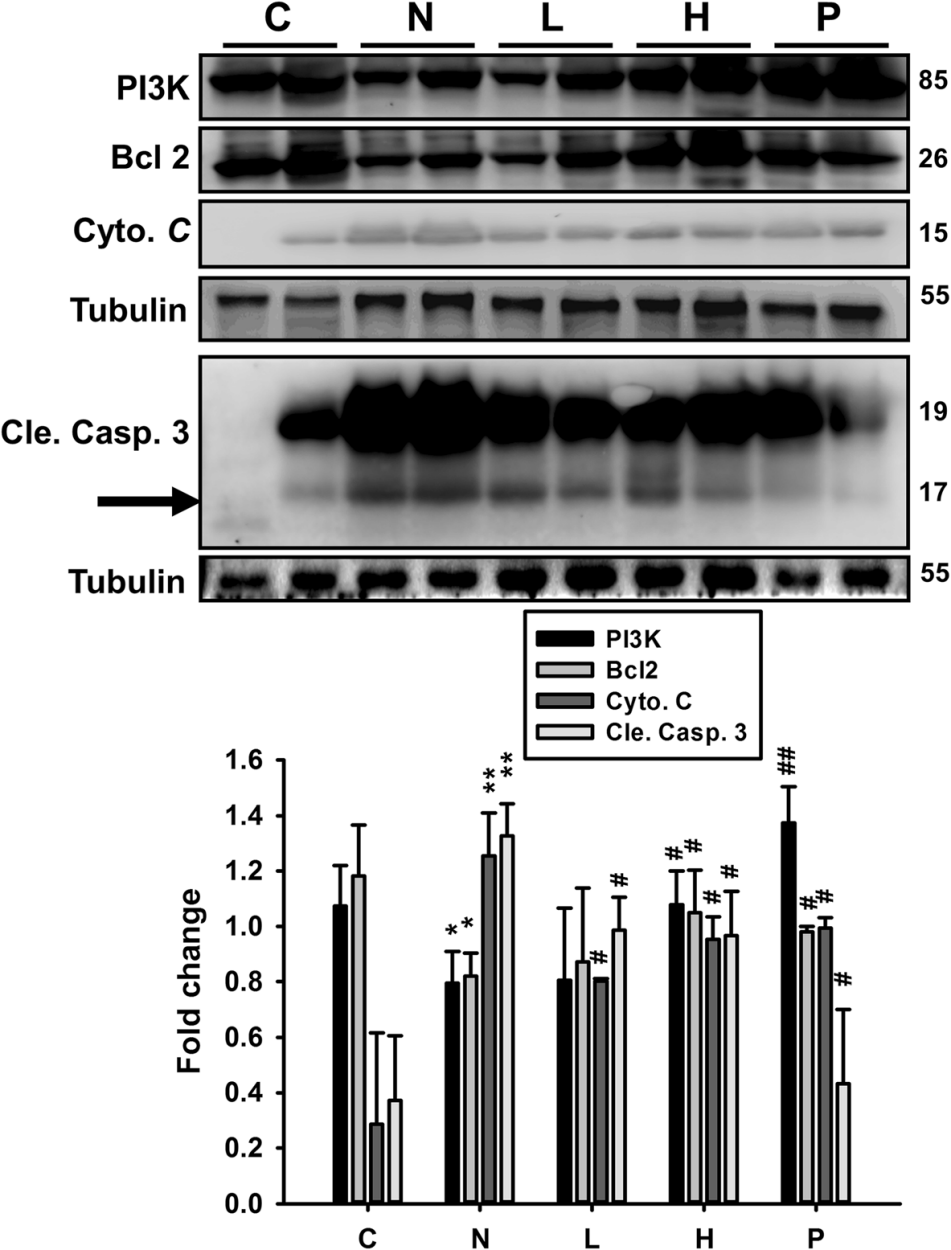
STZ-NA-induced apoptosis and survival pathway signaling analysis in liver tissues. All protein samples from each rat group were analyzed by western blotting (*n* = 6). The cell survival proteins included p-PI3K, p-Akt and Bcl2, and apoptosis proteins included Cyto. *c* and Cle. Casp. 3 in the control rats, STZ-NA rats, and treatment group rats. The protein expression folds were normalized with tubulin. C: control; N: STZ-NA + HFD; L: *Glossogyne tenuifolia* low dose (50 mg/kg); H: *Glossogyne tenuifolia* high dose (150 mg/kg); P: acarbose (positive control). Data represent mean ± SEM. *p < 0.05, **p < 0.01 compared with the C group; #P < 0.05, ##P < 0.01 when compared with the N group

To investigate the role of GT extracts on survival proteins in STZ-NA-induced changes in rats, the expression levels of proteins involv3ed in survival and apoptosis in the liver tissue from each group after 4 weeks of the given medications were analyzed by western blotting (Fig. 3). The expression levels of survival proteins p-PI3K and Bcl-2 were down-regulated, while there was an elevation in pro-apoptotic protein levels such as Cytochrome *c*, Cleaved Caspase 3 in STZ-NA-affected rat livers. After a 4-week treatment, we found a significant (*P* < 0.05) decrease in apoptosis protein expression levels, and the levels of survival proteins were elevated in liver tissues. However, in the mice groups that received GT extracts, the levels of survival protein expression were even more significantly increased and the levels of apoptosis proteins were suppressed.

### Effects of GT extracts on protein expression of hepatomegaly and fibrosis pathway in STZ-NA-affected diabetic rat livers

**Fig. 4:**
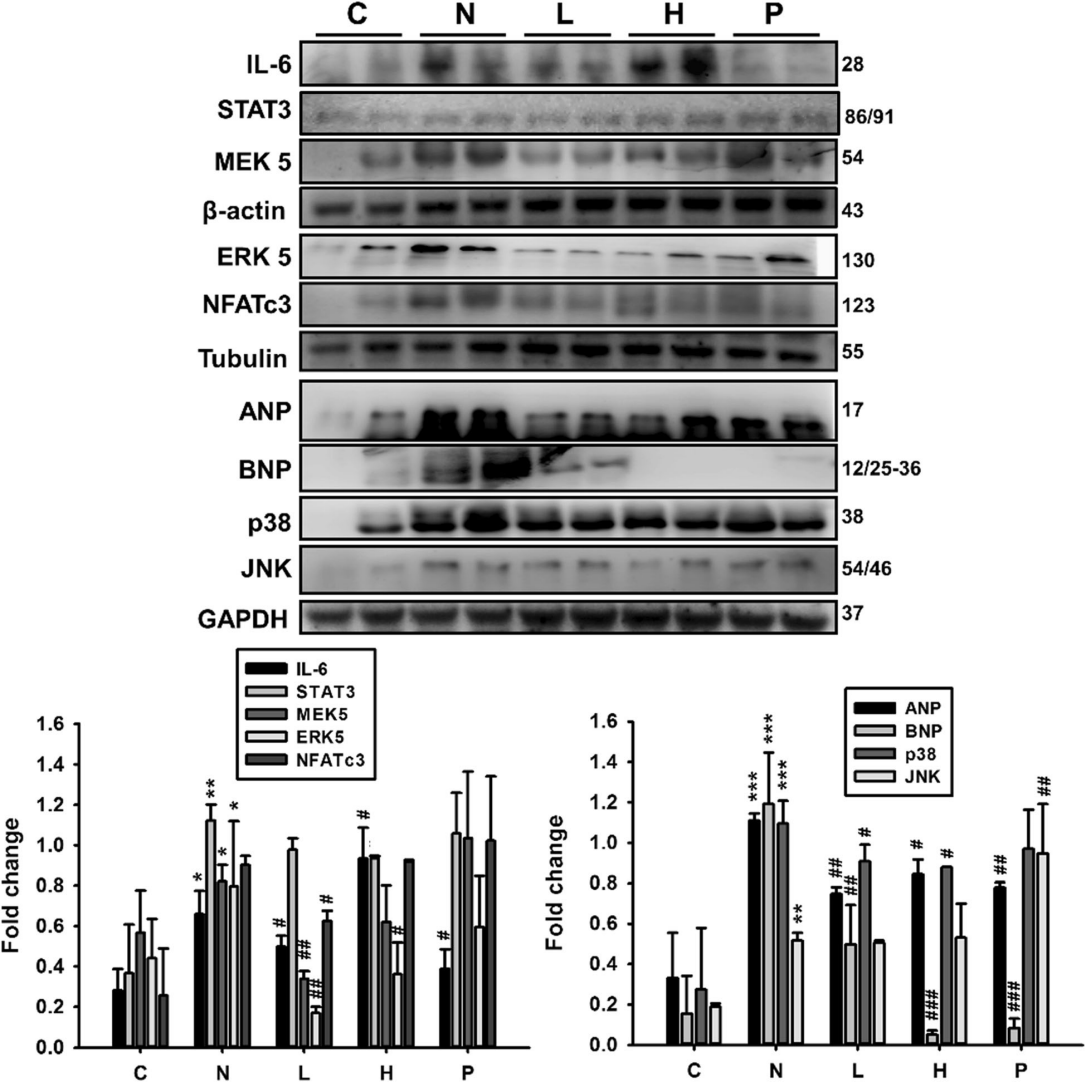
Effects of GT extracts on hypertrophy marker proteins in STZ-NA-induced changes in rat liver tissues. The levels of hypertrophy proteins IL-6, STAT3, MEK5, ERK5, NFATc3, p-p38 and p-JNK in the control rats, STZ-NA rats, and treatment group rats are shown. Data (*n* = 6) represent the mean ± SEM. *p < 0.05, **p < 0.01, ***p < 0.001 compared with the C group; #*P* < 0.05, ##*P* < 0.01 and ###*P* < 0.001 when compared with the N group. C: control; N: STZ-NA + HFD; L: *Glossogyne tenuifolia* low dose (50 mg/kg); H: *Glossogyne tenuifolia* high dose (150 mg/kg); P: acarbose (positive control)

**Fig. 5:**
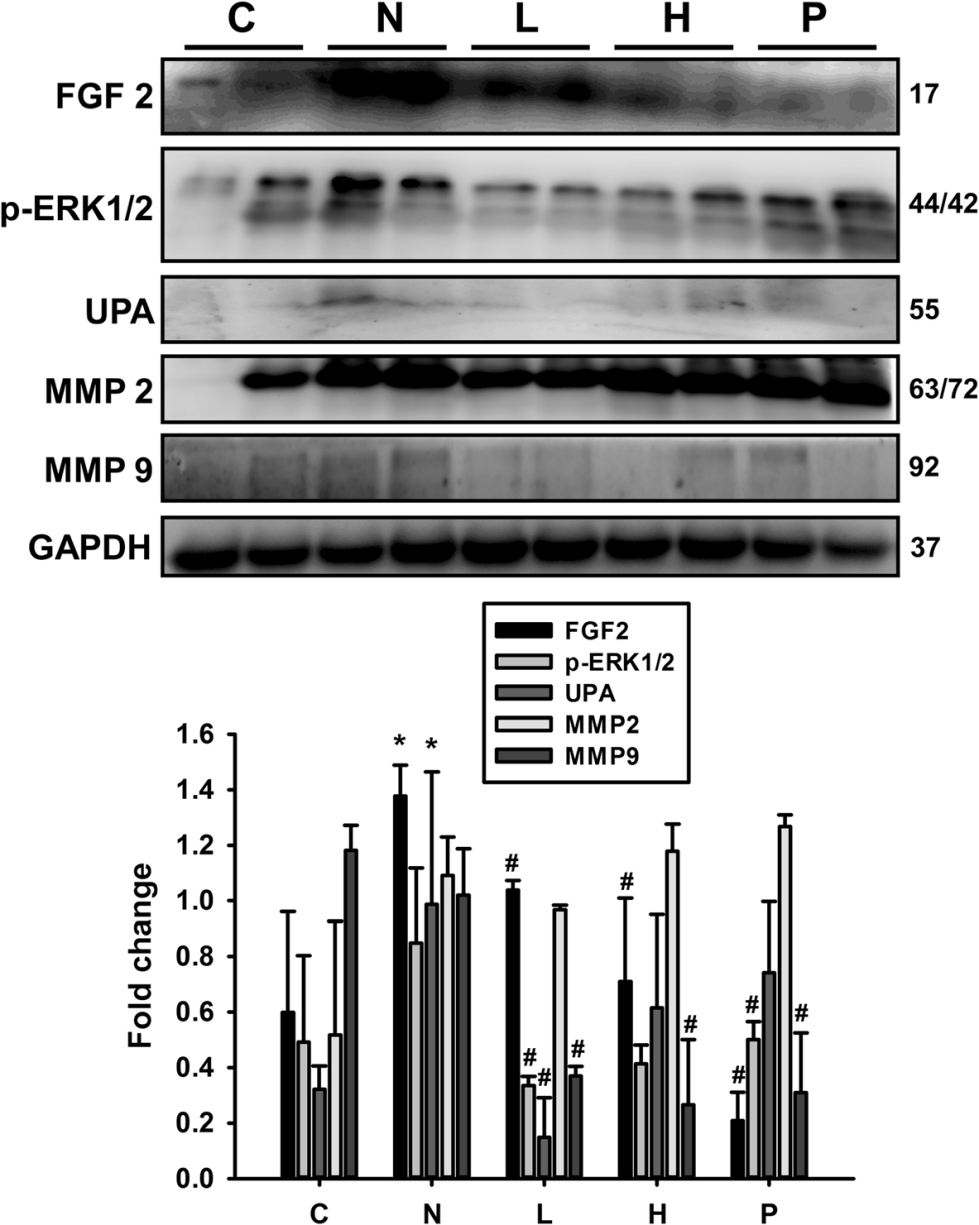
Effects of GT extracts on fibrosis marker proteins in STZ-NA-induced changes in rat liver tissues. The levels of hypertrophy protein FGF2, pERK 1/2, UPA, MMP2 and MMP9 in the control rats, STZ-NA rats, and treatment group rats are shown. Data (*n* = 6) represent the mean ± SEM. *p < 0.05 compared with the C group; #P < 0.05 when compared with the N group. C: control; N: STZ-NA + HFD; L: *Glossogyne tenuifolia* low dose (50 mg/kg); H: *Glossogyne tenuifolia* high dose (150 mg/kg); P: acarbose (positive control)

The protein expression levels in the DM rat liver tissues, analyzed by western blotting, showed that the occurrence of fatty liver in rats is associated with increased levels of the hypertrophy markers activated IL-6, STAT3, MEK5, ERK5, NFATc3, ANP, BNP, p-p38 and p-JNK (Fig. 4) and elevations in fibrosis protein levels such as FGF2, pERK 1/2, UPA, MMP2 and MMP9 (Fig. 5). However, in the DM rat groups administered with GT extracts, these markers were significantly (*P* < 0.05) suppressed (Figs. 4 and 5). The causes of chronic liver disease is generally multiple and diverse, from obesity to insulin resistance, inflammation and even oxidative stress. Our results indicate that STZ-NA treatment increases hepatomegaly and fibrosis in rat livers that was regulated when treated with GT extracts.

### GT extracts protects rats against STZ-NA-induced hyperlipidemia in liver

**Fig. 6:**
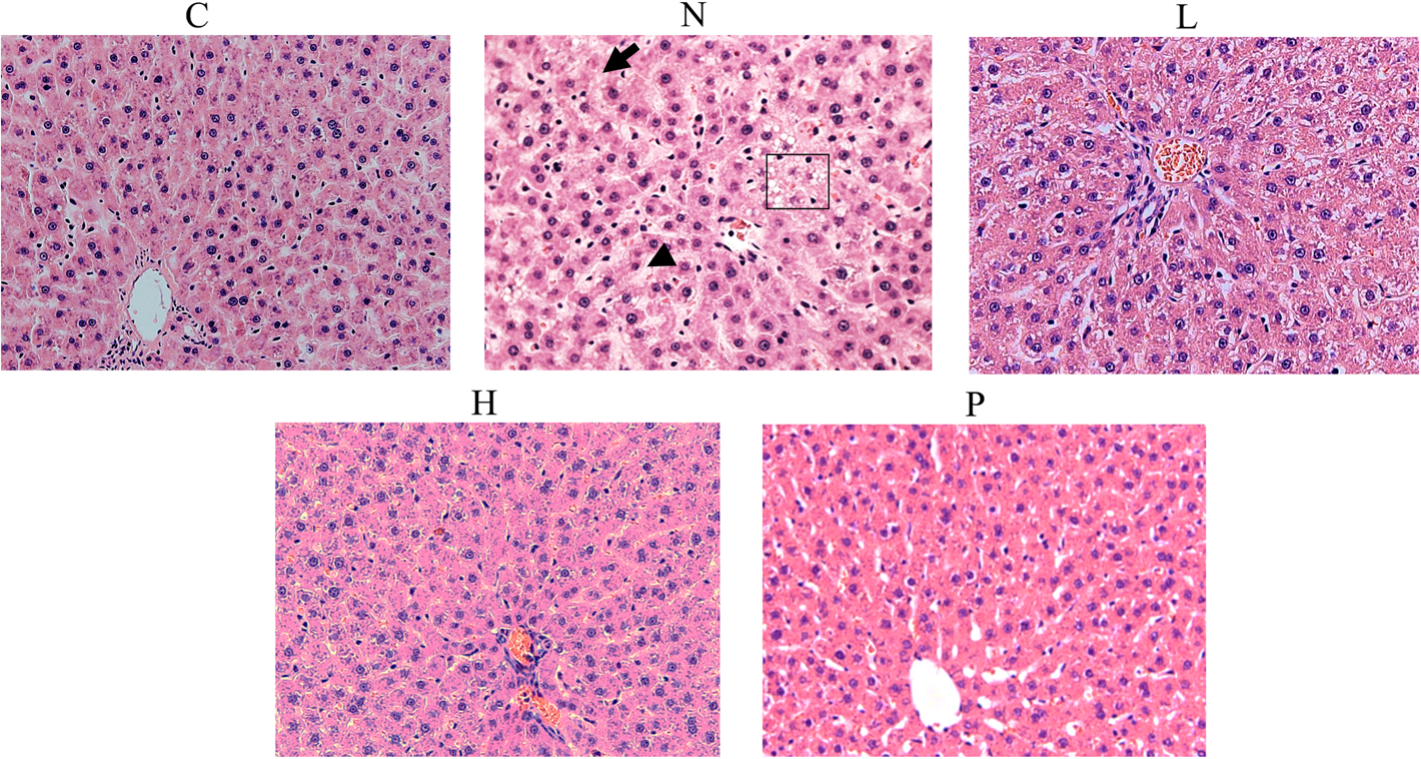
Hematoxylin and eosin (H&E) staining of tissue sections showing the liver tissue architecture and lipid droplets. Histopathological analysis of hepatic sections of control rats, STZ-NA rats, and treatment group rats was performed. Magnification 400x. C: control; N: STZ-NA + HFD; L: *Glossogyne tenuifolia* low dose (50 mg/kg); H: *Glossogyne tenuifolia* high dose (150 mg/kg); P: acarbose (positive control)

Hematoxylin and eosin (H&E) staining (Fig. 6) of liver tissue was performed to evaluate whether STZ-NA induced changes in the liver. In DM group the hepatic cords were arranged loosely and dilatation of sinusoids (arrow head) and hepatocyte vacuolation (arrow) were also observed. Enormous fat buildup (squared in box) in the rat livers in the HFD group animals was also observed by H&E-staining of the tissue. However, treatment with GT extracts efficiently attenuated the lipid accumulation in the liver tissues which was better than that observed in acarbose treatment groups.

### GT extracts protects rats against STZ-NA-induced liver fibrosis

**Fig. 7:**
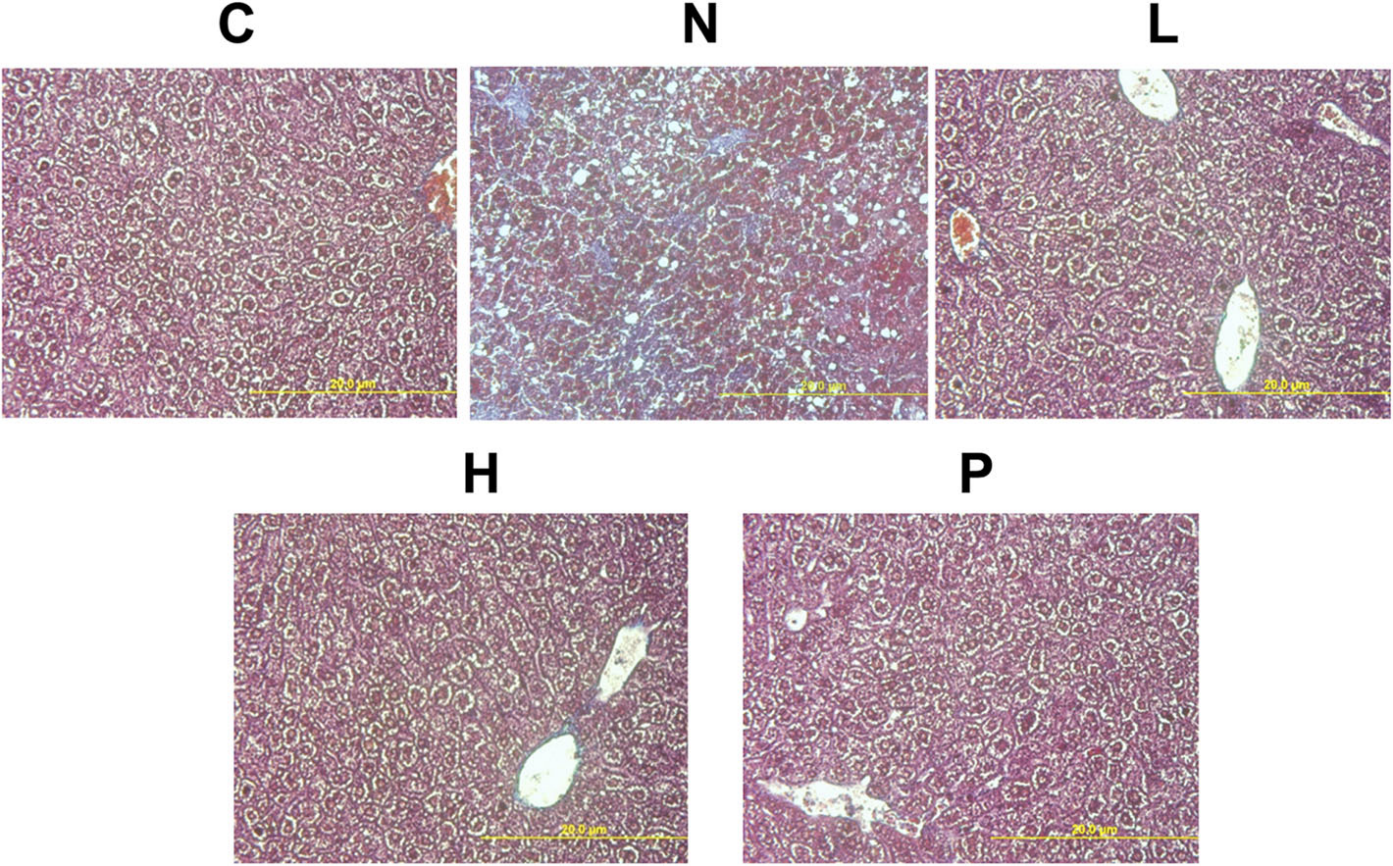
Effects of GT extracts on fibrosis in STZ-NA-induced changes in rat liver tissues. Masson’s trichrome staining in liver tissue sections from control rats, STZ-NA rats, and treatment group rats are shown. Magnification 400x. C: control; N: STZ-NA + HFD; L: *Glossogyne tenuifolia* low dose (50 mg/kg); H: *Glossogyne tenuifolia* high dose (150 mg/kg); P: acarbose (positive control)

Masson’s trichrome staining of liver tissue sections showed the effects of GT extracts on liver fibrosis in DM rats fed with HFD (Fig. 7). The DM liver tissue sections showed clear blue stain signifying collagen accumulation. However, GT treatment showed effective reduction in the fibrosis effects in STZ-NA administered rats. The results also show that GT extracts are more effective than the acarbose in inhibiting DM-associated liver fibrosis in HFD fed rat livers.

## Discussion

Diabetes mellitus (DM) is one of the leading cause of death caused by noncommunicable diseases in the world [30]. Increased levels of blood glucose level, a major characteristic of DM, cause oxidative stress and is associated with a variety of DM complications [31, 32]. The incidence of type II diabetes with hyperglycemia is increasing worldwide; it is a heterogeneous disease with progressive decline in insulin sensitivity causing pancreatic β-cell dysfunction [33, 34]. The pathogenesis of defective glucose transport systems plays a key role in peripheral insulin resistance. The balance of glucose homeostasis and clearance of postprandial glucose load are very important processes in the target tissue [35, 36].

NAFLD is an epidemic disease that is closely related to this metabolic syndrome and insulin resistance, and the prevalence of diabetes mellitus is more than 70% [37]. Accumulating evidences suggests the association between NAFLD and metabolic syndromes, and this association is prevalent in obese or diabetic patients who are also at high risk of liver fibrosis and cirrhosis [16, 38]. A recent study shows that the severity of NAFLD may partially determine cardiovascular risk among diabetic individuals [38, 39].

Two well-known mechanism leads to apoptotic response to different stresses [40]. The intrinsic pathway of apoptosis causes damage to mitochondrial membrane potential and results in the release of cytochrome *c* to induce caspase 9 [41, 42]. Bcl-2 is an anti-apoptotic that served as a crucial marker for the apoptosis event [43]. Our results show that the proteins of the intrinsic apoptosis are elevated in HFD fed DM rats but were attenuated when administered with GT extracts.

Subsequent to apoptosis or liver damage, hepatic stellate cells migrate to the site of apoptosis in order eliminate apoptotic bodies. The process involves hepatic stellate cells triggers excessive accumulation of extracellular matrix which may lead scaring in the liver [11]. This results in the progression of hepatic fibrosis that is generally associated with increasing levels of certain MMPs.

The effects of the GT extracts in STZ-NA rats were confirmed by analyzing the protein expression and by histopathological approach. Oral administration of GT extract also suppressed the hepatic apoptosis, and fibrosis-related proteins induced by STA-NA in HFD fed rats. Our results therefore indicate that GT extracts potentially attenuate hepatic lipid accumulation and displays anti-apoptosis, and and-fibrosis effects in STZ-NA-induced diabetic rats. GT extracts may have therapeutic potential in the amelioration of NAFLD liver damage.

Many extracts from herbs are known to avert drug-induced acute liver failure via the mediation of antioxidant effects [16, 44]. Therefore, the study of these drugs from traditional medicinal plants has become more and more important, and research is searching for new safe and effective drugs for the treatment of metabolic syndromes. Our results show the hepatoprotective effects of GT extracts against STZ-NA-induced hepatotoxicity in mice. GT extracts are known to contain oleanic acid, luteolin-7-glucoside and luteolin and has been correlated for their antioxidant potential. Free radical scavenging potential of flavonoids are potentially to their structural properties and depend on the characteristics of the radical. Luteolin exhibit higher superoxide and hydroxyl radical scavenging effects than its glycan luteolin-7-glucoside [45]. Luteolin administration is known to reduce liver fibrosis associated MMP-2 and MMP-9 induced by Pentylentetrazol toxicity [46]. Luteolin is also known to alleviate obesity associated NAFLD in diabetic mice by suppressing conversion of carbohydrates to TG [47]. Therefore the hepatoprotective effects of GT extract could be attributed to the presence of Luteolin.

## Conclusion

In conclusion, the results of this study demonstrated that in STZ-NA-induced diabetic rats, GT extracts might be effective in decreasing hepatocyte apoptosis and hypertrophy as well as attenuating fibrosis, which can be attributed to increases in antioxidant capacity and improved insulin resistance. GT extracts can also inhibit lipogenesis and lipid accumulations in the liver. The complementary actions of GT extracts may be beneficial for liver protection. Although, in addition to this results, there are various reports on the anti-inflammation, hypoglycemic, cyto-protective effects of GT and its bioactive constituents, further systematic investigations on the effects and mechanism of GT and its possible toxicity should be performed to encourage its potential application. Our future studies, will cover those aspects.

## Data Availability

The datasets used and/or analysed during the current study are available from the corresponding author on reasonable request.

## Abbreviations

DM: Diabetes mellitus
GT: *Glossogyne tenuifolia*
HFD: High-fat diet
NAFLD: Non-alcoholic fatty liver disease
STZ-NA: Streptozotocin-Nicotinamide
T2D: Type 2 diabetes mellitus
